# Solutions of Detour Distance Graph Equations

**DOI:** 10.3390/s22218440

**Published:** 2022-11-02

**Authors:** S. Celine Prabha, M. Palanivel, S. Amutha, N. Anbazhagan, Woong Cho, Hyoung-Kyu Song, Gyanendra Prasad Joshi, Hyeonjoon Moon

**Affiliations:** 1 Department of Mathematics, RMD Engineering College, Chennai 601 206, Tamil Nadu, India; 2 Department of Mathematics, Mepco Schlenk Engineering College, Sivakasi 626 005, Tamil Nadu, India; 3 Ramanujan Center for Higher Mathematics, Alagappa University, Karaikudi 630003, India; 4 Department of Mathematics, Alagappa University, Karaikudi 630003, India; 5 Department of Software Convergence, Daegu Catholic University, Gyeongsan 38430, Korea; 6 Department of Information and Communication Engineering and Convergence Engineering for Intelligent Drone, Sejong University, Seoul 05006, Korea; 7 Department of Computer Science and Engineering, Sejong University, Seoul 05006, Korea

**Keywords:** distance graphs, two-distance graphs, three-distance graphs, antipodal graph, cycle, line graph

## Abstract

Graph theory is a useful mathematical structure used to model pairwise relations between sensor nodes in wireless sensor networks. Graph equations are nothing but equations in which the unknown factors are graphs. Many problems and results in graph theory can be formulated in terms of graph equations. In this paper, we solved some graph equations of detour two-distance graphs, detour three-distance graphs, detour antipodal graphs involving with the line graphs.

## 1. Introduction

The recent rapid growth in the Internet of things has necessitated the development of new approaches to persistent issues in wireless sensor networks. These issues include minimum obstacles in the end-to-end communication path, location accuracy, latency, and delay, among others. These problems can be mitigated by using the distance graph to create local algorithms, i.e., algorithms with minimum communication rounds. We study distance graph applications in wireless sensor networks with a focus on minimum path obstacles and high localization accuracy.

A wireless sensor network (WSN) is a network of tiny wireless sensors that can sense a parameter of interest. The sensed data is forwarded to a base station through the formed ad hoc network of sensor nodes. There are many application areas of WSNs, including M2M communication and the Internet of Things (IoT). WSNs are a fundamental building block of smart homes, smart workplaces, and smart cities, among others. It has a lot of other essential purposes for modern technology, such as scientific research, rescue operations, and scientific discoveries. As sensors are a power constraint tiny devices, energy conservation for extending the network’s lifetime is a challenging issue. A wireless sensor network’s lifetime heavily depends on innovative schemes that mitigate energy consumption. The distance graph is used to form and localize an ad hoc network of sensor nodes so that sensed data can be forwarded to the base station with little energy cost.

Therefore, finding the solutions to these graph equations is essential. A lot of research has been done by many researchers during the past fifty years, and their results have made a significant contribution in graph theory.

Let *X* be a nontrivial finite connected graph. Every graph *X* with detour distance *D* defines a metric space. The *n*-distance graph of *X*, denoted by $T_{n}\left(X\right)$, is the graph with $V(T_{n}\left(X\right))=V\left(X\right)$ in which two vertices *u* and *v* are adjacent in $T_{n}\left(X\right)$ if and only if $d_{X}\left(u,v\right)=n$. Furthermore, for each set S⊆dist(V(X);D), the detour distance graph $\Gamma_{D}\left(V\left(X\right),S\right)$ is the graph having V(X) as its vertex set and two vertices *u* and *v* in this graph are adjacent if and only if D(u,v)∈S. We denote this graph simply by D(X,S). The graph $D_{n}\left(X\right):=D\left(X,\left\{n\right\}\right)$ is called the *detour n-distance graph* of *X*. If *n* equals the detour diameter of *X*, then this graph is called the *detour antipodal graph* of *X*, which is denoted by DA(G). A graph *X* is said to be a *detour self n-distance graph* if $D_{n}\left(X\right)≅X$.

The line graph L(G) of a graph *G* is the graph whose vertices correspond to the edges of *G* and wherein two vertices are adjacent in L(G) if and best if the corresponding edges are adjacent in *G*.

In this work, we consider the detour distance graphs. Specifically, we solve the graph equations involving detour two-distance graphs, detour three-distance graphs, detour antipodal distance graphs, line graphs, and the complement of graphs. In addition, we solve some equations involving two-distance graphs, three-distance graphs, and antipodal distance graphs with the graphs mentioned earlier.

Harary, Heode, and Kedlacek first studied the two-distance graph. They investigated the connectedness of two-distance graphs. This graph and the relationship between the two-distance graph and line graph was further studied in [1,2,3,4,5,6,7,8,9]. In 2014, Ali Azimi and Mohammad D. X solved the graph equations $T_{2}\left(X\right)≅P_{n}$ and $T_{2}\left(X\right)≅C_{n}$. In 2015, Ramuel P. Ching and Garces gave three characterizations of two-distance graphs and found all the graphs *X* such that $T_{2}\left(X\right)≅kP_{2}$ or $K_{m}\cup K_{n}$, which can be found in [10]. S. K Simic and some mathematicians solved some graph equations of line graphs, which can be found in [11,12,13,14,15,16,17]. In 2017, R. Rajkumar and S. Celine Prabha solved some graph equations of two-distance, three-distance and *n*-distance graph equations, which can be found in [18,19,20]. In 2018, R. Rajkumar and S. Celine Prabha found the characterization of the distance graph of a path which was described in [21].

Motivated by the results listed above, we solved some graph equations of distance graphs and line graphs. Other graph-theoretic terms and notations that are not explicitly defined here can be found in [2].

## 2. Solutions of Graph Equations of Detour Two-Distance Graphs and Line Graphs

First, we consider some graph equations of type $D_{2}\left(G\right)≅G_{1}\cup G_{2}$, where $G_{1}$ and $G_{2}$ are given graphs and investigate the solution *G* of these equations.

The main result of solving graph equations involving detour two-distance graphs we prove in this section is the following:

**Theorem** **1.** *Let G be a graph. Then,*

1. *$D_{2}\left(G\right)≅\bar{L(G)}$ if and only if $G≅C_{4}$;*
2. *$D_{2}\left(L\left(G\right)\right)≅\bar{G}$ if and only if $G≅C_{4}$;*
3. *$D_{2}\left(D_{2}\left(L\left(G\right)\right)\right)≇\bar{G}$;*
4. *$T_{2}\left(D_{2}\left(L\left(G\right)\right)\right)≅\bar{G}$ if and only if $G≅C_{3}$;*
5. *$T_{2}\left(D_{2}\left(G\right)\right)≅\bar{L(G)}$ if and only if $G≅C_{3}$;*
6. *If G is connected, then*
  (a) *$D_{2}\left(G\right)≅L\left(G\right)$ if and only if $G≅C_{3}$;*
  (b) *$D_{2}\left(L\left(G\right)\right)≅G$ if and only if $G≅C_{3}$;*
  (c) *$D_{2}\left(\bar{L(G)}\right)≇G$;*
  (d) *$D_{2}\left(\bar{L(G)}\right)≅\bar{G}$ if and only if $G≅C_{3}$;*
  (e) *$D_{2}\left(D_{2}\left(L\left(G\right)\right)\right)≅G$ if and only if $G≅C_{3}$;*
  (f) *$T_{2}\left(D_{2}\left(L\left(G\right)\right)\right)≇G$;*
  (g) *$T_{2}\left(D_{2}\left(\bar{L(G)}\right)\right)≇G$;*
  (h) *$T_{2}\left(D_{2}\left(\bar{L(G)}\right)\right)≅\bar{G}$ if and only if $G≅C_{3}$;*
  (i) *$D_{2}\left(D_{2}\left(G\right)\right)≅L\left(G\right)$ if and only if $G≅C_{3}$.*

**Lemma** **1.** *Let n≥3 be an integer. Then,*

$$D_{2}\left(C_{n}\right)≅\left\{\begin{array}{cc} C_{3}, & \mathrm{if}\;n=3;\\ 2K_{2}, & \mathrm{if}\;n=4;\\ \bar{K_{n}}, & \mathrm{if}\;n\geq 5. \end{array}\right.$$

**Proof.**  Clearly $D_{2}\left(C_{3}\right)≅C_{3}$ and $D_{2}\left(C_{4}\right)≅2K_{2}$. If n≥5, then the detour distance between any two vertices of $C_{n}$ is at least three, so $D_{2}\left(C_{n}\right)≅\bar{K_{n}}$. □

**Proposition** **1.** *Let n≥3 be an integer. Then,*

1. *$D_{2}\left(C_{n}\right)≅L\left(C_{n}\right)$, $D_{2}\left(L\left(C_{n}\right)\right)≅C_{n}$, $D_{2}\left(\bar{L(C_{n})}\right)≅\bar{C_{n}}$,*
*$D_{2}\left(D_{2}\left(L\left(C_{n}\right)\right)\right)≅C_{n}$, $T_{2}\left(D_{2}\left(L\left(C_{n}\right)\right)\right)≅\bar{C_{n}}$,*
*$T_{2}\left(D_{2}\left(\bar{L(C_{n})}\right)\right)≅\bar{C_{n}}$, $D_{2}\left(D_{2}\left(C_{n}\right)\right)≅L\left(C_{n}\right)$ and $T_{2}\left(D_{2}\left(C_{n}\right)\right)≅\bar{L(C_{n})}$ if and only if n=3;*
2. *$D_{2}\left(C_{n}\right)≅\bar{L(C_{n})}$, $D_{2}\left(L\left(C_{n}\right)\right)≅\bar{C_{n}}$ if and only if n=4;*
3. *$D_{2}\left(\bar{L(C_{n})}\right)≇C_{n}$, $D_{2}\left(D_{2}\left(L\left(C_{n}\right)\right)\right)≇\bar{C_{n}}$, $T_{2}\left(D_{2}\left(L\left(C_{n}\right)\right)\right)≇C_{n}$,*
*$T_{2}\left(D_{2}\left(\bar{L(C_{n})}\right)\right)≇C_{n}$ and $D_{2}\left(C_{n}\right)≇L\left(\bar{C_{n}}\right)$ for all n≥3.*

**Proof.**  Clearly $L\left(C_{n}\right)≅C_{n}$. Note that $\bar{C_{3}}≅\bar{K_{3}}$ and $\bar{C_{4}}≅2K_{2}$. Furthermore, if n≥5, then $\bar{C_{n}}$ is connected and is (n−3)-regular. Therefore, the proof follows from Lemma 1. □

**Proposition** **2.** *Let G be a graph.*

1. *If G is connected non-unicyclic, then $D_{2}\left(G\right)≇\bar{L(G)}$, $D_{2}\left(L\left(G\right)\right)≇\bar{G}$, $D_{2}\left(D_{2}\left(L\left(G\right)\right)\right)≇\bar{G}$, $T_{2}\left(D_{2}\left(L\left(G\right)\right)\right)≇\bar{G}$, $T_{2}\left(D_{2}\left(G\right)\right)≇\bar{L(G)}$, $D_{2}\left(G\right)≇L\left(G\right)$, $D_{2}\left(G\right)≇L\left(\bar{G}\right)$,*
*$D_{2}\left(L\left(G\right)\right)≇G$, $D_{2}\left(\bar{L(G)}\right)≇G$, $D_{2}\left(\bar{L(G)}\right)≇\bar{G}$, $D_{2}\left(D_{2}\left(L\left(G\right)\right)\right)≇G$, $T_{2}\left(D_{2}\left(L\left(G\right)\right)\right)≇G$, $T_{2}\left(D_{2}\left(\bar{L(G)}\right)\right)≇G$, $T_{2}\left(D_{2}\left(\bar{L(G)}\right)\right)≇\bar{G}$ and $D_{2}\left(D_{2}\left(G\right)\right)≇L\left(G\right)$.*
2. *If G is disconnected, then $T_{2}\left(D_{2}\left(L\left(G\right)\right)\right)≇\bar{G}$ and $T_{2}\left(D_{2}\left(G\right)\right)≇\bar{L(G)}$.*

**Proof.** 

1. Let *G* be connected non-unicyclic. Suppose that $D_{2}\left(G\right)≅\bar{L(G)}$. Then, we have |V(G)|=|E(G)|, so *G* is unicyclic, since *G* is connected, which is a contradiction to our assumption that *G* is non-unicyclic. Thus, $D_{2}\left(G\right)≇\bar{L(G)}$. The proof of the rest of the cases are similar to the above.
2. Let *G* be disconnected. Then, $T_{2}\left(D_{2}\left(L\left(G\right)\right)\right)$, $T_{2}(D_{2}\left(G\right)$ are disconnected; $\bar{G}$ and $\bar{L(G)}$ are connected. Combining these pieces of information, we get the result.

□

**Proposition** **3.** *Let G be a connected unicyclic graph but not a cycle. Then,*

1. *$D_{2}\left(G\right)≇L\left(G\right)$;*
2. *$D_{2}\left(G\right)≇\bar{L(G)}$;*
3. *$D_{2}\left(L\left(G\right)\right)≇G$;*
4. *$D_{2}\left(L\left(G\right)\right)≇\bar{G}$;*
5. *$D_{2}\left(\bar{L(G)}\right)≇G$;*
6. *$D_{2}\left(\bar{L(G)}\right)≇\bar{G}$;*
7. *$D_{2}\left(D_{2}\left(L\left(G\right)\right)\right)≇G$;*
8. *$D_{2}\left(D_{2}\left(L\left(G\right)\right)\right)≇\bar{G}$;*
9. *$T_{2}\left(D_{2}\left(L\left(G\right)\right)\right)≇G$;*
10. *$T_{2}\left(D_{2}\left(L\left(G\right)\right)\right)≇\bar{G}$;*
11. *$T_{2}\left(D_{2}\left(\bar{L(G)}\right)\right)≇G$;*
12. *$T_{2}\left(D_{2}\left(\bar{L(G)}\right)\right)≇\bar{G}$;*
13. *$D_{2}\left(D_{2}\left(G\right)\right)≇L\left(G\right)$;*
14. *$T_{2}\left(D_{2}\left(G\right)\right)≇\bar{L(G)}$.*

**Proof.** 

1. Let $C_{k},\;k\geq 3$ be the induced cycle of *G*. Let this cycle be $v_{1}-v_{2}-\cdots -v_{k}-v_{1}$. Let *v* be a vertex in *G* which is not in the cycle of *G*. Without loss of generality, we may assume that *v* is adjacent to $v_{1}$. Then, $D(v,v_{1})\geq 3$ is in $D_{2}\left(G\right)$, so $D_{2}\left(G\right)$ is disconnected. However, L(G) is connected. Therefore, $D_{2}\left(G\right)≇L\left(G\right)$.
2. If $G≅C_{3}\left(r,0,0\right)$ or $C_{3}\left(r,s,0\right),\;r,\;s\geq 1$, then $D_{2}\left(G\right)$ has two components. However, $\bar{L(G)}$ has three components. Thus, $D_{2}\left(\left(G\right)\right)≇\bar{L(G)}$.
  If *G* is the graph other than these graphs, then by the argument of part (a), $D_{2}\left(G\right)$ is disconnected. Hence, $\bar{L(G)}$ is connected. Therefore, $D_{2}\left(G\right)≇\bar{L(G)}$.
3. By the structure of *G*, the graph L(G) is connected non-unicyclic. Let the maximum length among all the cycles in L(G) be *k*. Clearly k≥4. If k≥5, then any vertex in such a cycle is isolated in $D_{2}\left(L\left(G\right)\right)$. If k=4, then *G* is the graph obtained from $K_{3}$ by adding a pendent edge to any of its vertex. In this case, $D_{2}\left(L\left(G\right)\right)$ is disconnected. Thus, $D_{2}\left(L\left(G\right)\right)≇G$.
4. By part (c), $D_{2}\left(L\left(G\right)\right)$ is disconnected. However, $\bar{G}$ is connected except for the graph $C_{3}\left(r,0,0\right),\;r\geq 1$. Therefore, $D_{2}\left(L\left(G\right)\right)≇\bar{G}$. If $G≅C_{3}\left(r,0,0\right),\;r\geq 1$, then $D_{2}\left(L\left(G\right)\right)$ has two isolated vertices. However, $\bar{G}$ has exactly one isolated vertex. Thus, $D_{2}\left(L\left(G\right)\right)≇\bar{G}$.
5. If $G≅C_{3}\left(r,0,0\right)$ or $C_{3}\left(r,s,0\right),\;r,\;s\geq 2$, then $\bar{L(G)}$ is disconnected and so $D_{2}\left(\bar{L(G)}\right)$ is disconnected. Thus, $D_{2}\left(\bar{L(G)}\right)≇G$.
  If *G* is the graph other than these graphs, then the maximum length among all the cycles in $\bar{L(G)}$ is at least six. Then, any vertex in such a cycle is isolated in $D_{2}\left(\bar{L(G)}\right)$. Therefore, $D_{2}\left(\bar{L(G)}\right)≇G$.
6. By part (e), $D_{2}\left(\bar{L(G)}\right)$ is disconnected. Thus, by a similar argument as in part (d), we get $D_{2}\left(\bar{L(G)}\right)≇\bar{G}$.
7. By part (c), $D_{2}\left(L\left(G\right)\right)$ is disconnected. Therefore, $D_{2}\left(D_{2}\left(L\left(G\right)\right)\right)$ is disconnected and so $D_{2}\left(D_{2}\left(L\left(G\right)\right)\right)≇G$.
8. If *G* is the graph other than the graph $C_{3}\left(r,0,0\right),\;r\geq 1$, then $\bar{G}$ is connected. By part (c), $D_{2}\left(L\left(G\right)\right)$ is disconnected. Thus, $D_{2}\left(D_{2}\left(L\left(G\right)\right)\right)$ is disconnected and so $D_{2}\left(D_{2}\left(L\left(G\right)\right)\right)≇\bar{G}$. If $G≅C_{3}\left(r,0,0\right),r\geq 1$, then $D_{2}\left(D_{2}\left(L\left(G\right)\right)\right)$ contains at least two isolated vertices. However, $\bar{G}$ has exactly one isolated vertex. Thus, $D_{2}\left(D_{2}\left(L\left(G\right)\right)\right)≇\bar{G}$.
9. By part (c), $D_{2}\left(L\left(G\right)\right)$ is disconnected. Therefore, $T_{2}\left(D_{2}\left(L\left(G\right)\right)\right)$ is disconnected and so $T_{2}\left(D_{2}\left(L\left(G\right)\right)\right)≇G$.
10. The proof is similar to the proof of part (h), since $T_{2}\left(D_{2}\left(L\left(G\right)\right)\right)$ is also disconnected.
11. By part (e), $D_{2}\left(\bar{L(G)}\right)$ is disconnected. Thus, $T_{2}\left(D_{2}\left(\bar{L(G)}\right)\right)$ is also disconnected. Consequently, we get $T_{2}\left(D_{2}\left(\bar{L(G)}\right)\right)≇G$.
12. By part (e), $D_{2}\left(\bar{L(G)}\right)$ is disconnected. However, $\bar{G}$ is connected except for the graph $C_{3}\left(r,0,0\right),\;r\geq 1$. So $T_{2}\left(D_{2}\left(\bar{L(G)}\right)\right)≇\bar{G}$. If $G≅C_{3}\left(r,0,0\right),\;r\geq 1$, then $T_{2}\left(D_{2}\left(\bar{L(G)}\right)\right)$ has at least two isolated vertices but $\bar{G}$ has exactly one isolated vertex.
  Therefore, $T_{2}\left(D_{2}\left(\bar{L(G)}\right)\right)≇\bar{G}$.
13. By part (a) $D_{2}\left(G\right)$ is disconnected. Thus, L(G) is connected and $D_{2}\left(D_{2}\left(G\right)\right)≇L\left(G\right)$.
14. If $G≅C_{3}\left(r,0,0\right)$ or $C_{3}\left(r,s,0\right),\;r,\;s\geq 1$, then $D_{2}\left(G\right)$ has two components. However, $\bar{L(G)}$ has three components. Therefore, $T_{2}\left(D_{2}\left(G\right)\right)≇\bar{L(G)}$.
  If *G* is the graph other than these graphs, then by the argument of part (a), $D_{2}\left(G\right)$ is disconnected and so $T_{2}\left(D_{2}\left(L(G)\right)\right)$ is disconnected. Hence, $\bar{L(G)}$ is connected. Therefore, $T_{2}\left(D_{2}\left(L(G)\right)\right)≇\bar{L(G)}$.

□

Combining Propositions 1–3, we get the proof of Theorem 1.

## 3. Solutions of Graph Equations of Detour Three-Distance Graphs and Line Graphs

The main result on solving graph equations involving three-distance graphs we prove in this section is the following:

**Theorem** **2.** *Let G be a graph. Then,*

1. *$D_{3}\left(L\left(G\right)\right)≅\bar{G}$ if and only if $G≅C_{5}$;*
2. *$D_{3}\left(D_{3}\left(L\left(G\right)\right)\right)≅\bar{G}$ if and only if $G≅C_{5}$;*
3. *$T_{2}\left(D_{3}\left(L\left(G\right)\right)\right)≅\bar{G}$ if and only if $G≅C_{5}$ or $C_{4}$;*
4. *If G is connected, then*
  (a) *$D_{3}\left(L\left(G\right)\right)≅G$ if and only if $G≅C_{4}$ or $C_{5}$;*
  (b) *$D_{3}\left(\bar{L(G)}\right)≅G$ if and only if $G≅C_{5}$;*
  (c) *$D_{3}\left(\bar{L(G)}\right)≅\bar{G}$ if and only if $G≅C_{5}$;*
  (d) *$D_{3}\left(D_{3}\left(L\left(G\right)\right)\right)≅G$ if and only if $G≅C_{4}$ or $C_{5}$;*
  (e) *$T_{2}\left(D_{3}\left(L\left(G\right)\right)\right)≅G$ if and only if $G≅C_{5}$;*
  (f) *$T_{2}\left(D_{3}\left(\bar{L(G)}\right)\right)≅G$ if and only if $G≅C_{5}$;*
  (g) *$T_{2}\left(D_{3}\left(\bar{L(G)}\right)\right)≅\bar{G}$ if and only if $G≅C_{5}$.*

To prove the above theorem, we start with the following:

**Lemma** **2.** *Let n≥4 be an integer. Then,*

$$D_{3}\left(C_{n}\right)=\left\{\begin{array}{cc} C_{4}, & \mathrm{if}\;n=4;\\ C_{5}. & \mathrm{if}\;n=5;\\ 3K_{2}, & \mathrm{if}\;n=6;\\ \bar{K_{n}}, & \mathrm{if}\;n\geq 7. \end{array}\right.$$

**Proof.**  Clearly, $D_{3}\left(C_{4}\right)≅C_{4}$, $D_{3}\left(C_{5}\right)≅C_{5}$ and $D_{3}\left(C_{6}\right)≅3K_{2}$. If n≥7, then the detour distance between any two vertices of $C_{n}$ is at least four. Therefore, $D_{3}\left(C_{n}\right)≅\bar{K_{n}}$. □

**Proposition** **4.** *Let n≥4 be an integer. Then,*

1. *$D_{3}\left(\bar{L(C_{n})}\right)≅\bar{C_{n}}$, $T_{2}\left(D_{3}\left(\bar{L(C_{n})}\right)\right)≅\bar{C_{n}}$, $D_{3}\left(L\left(C_{n}\right)\right)≅\bar{C_{n}}$, $D_{3}\left(D_{3}\left(L\left(C_{n}\right)\right)\right)≅\bar{C_{n}}$, $T_{2}\left(D_{3}\left(L\left(C_{n}\right)\right)\right)≅C_{n}$, $T_{2}\left(D_{3}\left(\bar{L(C_{n})}\right)\right)≅C_{n}$ and $D_{3}\left(C_{n}\right)≅L\left(\bar{C_{n}}\right)$ if and only if n=5;*
2. *$D_{3}\left(L\left(C_{n}\right)\right)≅C_{n}$, $D_{3}\left(D_{3}\left(L\left(C_{n}\right)\right)\right)≅C_{n}$, $T_{2}\left(D_{3}\left(L\left(C_{n}\right)\right)\right)≅\bar{C_{n}}$, if and only if n=4,5;*
3. *$D_{3}\left(\bar{L(C_{n})}\right)≅C_{n}$ if and only if n=5.*

**Proof.**  Clearly, $L\left(C_{n}\right)≅C_{n}$. Note that $\bar{C_{3}}≅\bar{K_{3}}$ and $\bar{C_{4}}≅2K_{2}$. Furthermore, if n≥5, then $\bar{C_{n}}$ is connected and is (n−3)-regular. Thus, the proof follows from Lemma 2. □

**Proposition** **5.** *Let G be a graph.*

1. *If G is connected non-unicyclic, then $D_{3}\left(L\left(G\right)\right)≇\bar{G}$, $D_{3}\left(D_{3}\left(L\left(G\right)\right)\right)≇\bar{G}$,*
*$T_{2}\left(D_{3}\left(L\left(G\right)\right)\right)≇\bar{G}$, $D_{3}\left(L\left(G\right)\right)≇G$, $D_{3}\left(\bar{L(G)}\right)≇G$; $D_{3}\left(\bar{L(G)}\right)≇\bar{G}$, $D_{3}\left(D_{3}\left(L\left(G\right)\right)\right)≇G$,*
*$T_{2}\left(D_{3}\left(L\left(G\right)\right)\right)≇G$, $T_{2}\left(D_{3}\left(\bar{L(G)}\right)\right)≇G$ and $T_{2}\left(D_{3}\left(\bar{L(G)}\right)\right)≇\bar{G}$.*
2. *If G is disconnected, then $D_{3}\left(L\left(G\right)\right)≇\bar{G}$, $D_{3}\left(D_{3}\left(L\left(G\right)\right)\right)≇\bar{G}$ and $T_{2}\left(D_{3}\left(L\left(G\right)\right)\right)≇\bar{G}$.*

**Proof.** 

1. Let *G* be connected non-unicyclic. Suppose that $D_{3}\left(L\left(G\right)\right)≅\bar{G}$. Then, we have |V(G)|=|E(G)|, so *G* is unicyclic, since *G* is connected, which is a contradiction to our assumption that *G* is non-unicyclic. Thus, $D_{3}\left(L\left(G\right)\right)≇\bar{G}$. The proof of the rest of the cases are similar to the above.
2. Let *G* be disconnected. Then, $D_{3}\left(L\left(G\right)\right)$$D_{3}\left(D_{3}\left(L\left(G\right)\right)\right)$, $T_{2}\left(D_{3}\left(L\left(G\right)\right)\right)$ are disconnected and $\bar{G}$ is connected. Combining these pieces of information, we get the result.

□

**Proposition** **6.** *Let G be a connected unicyclic graph but not a cycle. Then,*

1. *$D_{3}\left(L\left(G\right)\right)≇G$;*
2. *$D_{3}\left(L\left(G\right)\right)≇\bar{G}$;*
3. *$D_{3}\left(\bar{L(G)}\right)≇G$;*
4. *$D_{3}\left(\bar{L(G)}\right)≇\bar{G}$;*
5. *$D_{3}\left(D_{3}\left(L\left(G\right)\right)\right)≇G$;*
6. *$D_{3}\left(D_{3}\left(L\left(G\right)\right)\right)≇\bar{G}$;*
7. *$T_{2}\left(D_{3}\left(L\left(G\right)\right)\right)≇G$;*
8. *$T_{2}\left(D_{3}\left(L\left(G\right)\right)\right)≇\bar{G}$;*
9. *$T_{2}\left(D_{3}\left(\bar{L(G)}\right)\right)≇G$;*
10. *$T_{2}\left(D_{3}\left(\bar{L(G)}\right)\right)≇\bar{G}$.*

**Proof.** 

1. By the structure of *G*, the graph L(G) is connected non-unicyclic. Let the maximum length among all the cycles in L(G) be *k*. Clearly, k≥4. If k≥7, then any vertex in such a cycle is isolated in $D_{3}\left(L\left(G\right)\right)$. If k=4,5,6, then $D_{3}\left(L\left(G\right)\right)≅C_{4}$, $C_{5}$ and $3K_{2}$, respectively. Thus, $D_{3}\left(L\left(G\right)\right)$ is disconnected and $D_{3}\left(L\left(G\right)\right)≇G$.
2. By part (a), $D_{3}\left(L\left(G\right)\right)$ is disconnected. However, $\bar{G}$ is connected except for the graph $C_{3}\left(r,0,,0\right),\;r\geq 1$. Thus, $D_{3}\left(L\left(G\right)\right)≇\bar{G}$. If $G≅C_{3}\left(r,0,,0\right),\;r\geq 1$, then $D_{3}\left(L\left(G\right)\right)$ has two isolated vertices. However, $\bar{G}$ has exactly one isolated vertex. Therefore, $D_{3}\left(L\left(G\right)\right)≇\bar{G}$.
3. If $G≅C_{3}\left(r,0,,0\right)$ or $C_{3}\left(r,s,0\right)$, r,s≥1, then $\bar{L(G)}$ is disconnected, so $D_{3}\left(\bar{L(G)}\right)$ is disconnected. Thus, $D_{3}\left(\bar{L(G)}\right)≇G$.
  If *G* is the graph other than these graphs, then the maximum length among all the cycles in $\bar{L(G)}$ is at least seven. Then, any vertex in such a cycle is isolated in $D_{3}\left(\bar{L(G)}\right)$. Thus, $D_{3}\left(\bar{L(G)}\right)≇G$.
4. By part (c), $D_{3}\left(\bar{L(G)}\right)$ is disconnected. Therefore, the rest of the proof is similar to part (b).
5. By part (a), $D_{3}\left(L\left(G\right)\right)$ is disconnected. Thus, $D_{3}\left(D_{3}\left(L\left(G\right)\right)\right)$ is disconnected and so $D_{3}\left(D_{3}\left(L\left(G\right)\right)\right)≇G$.

(f)- The proof is similar to the proof of part (b), since $D_{3}\left(D_{3}\left(L\left(G\right)\right)\right)$, $T_{2}\left(D_{3}\left(L\left(G\right)\right)\right)$ and
(h): $T_{3}\left(D_{3}\left(L\left(G\right)\right)\right)$ are disconnected.
(i)- The proof of part (i) and (j) are similar to the proof of parts (c) and (d), respectively,
(j): since $D_{3}\left(\bar{L(G)}\right)$ is disconnected.

□

Combining Propositions 4–6, we get the proof of Theorem 2.

## 4. Solutions of Graph Equations of Detour Antipodal Graphs and Line Graphs

The main result on solving graph equations involving detour antipodal graphs we prove in this section is the following:

**Theorem** **3.** *Let G be a graph. Then,*

1. *DA(G)≅L(G) if and only if $G≅C_{n}$ for some n≥3;*
2. *$\mathrm{DA}\left(G\right)≅\bar{L(G)}$ if and only if $G≅C_{5}$;*
3. *DA(L(G))≅G if and only if $G≅C_{n}$ for some n≥3;*
4. *$\mathrm{DA}\left(L\left(G\right)\right)≅\bar{G}$ if and only if $G≅C_{5}$;*
5. *$D_{2}\left(\mathrm{DA}\left(L\left(G\right)\right)\right)≅G$ if and only if $G≅C_{3}$;*
6. *$D_{2}\left(\mathrm{DA}\left(G\right)\right)≅G$ if and only if $G≅C_{3}$;*
7. *$D_{3}\left(\mathrm{DA}\left(L\left(G\right)\right)\right)≅G$ if and only if $G≅C_{4}$.*
8. *$D_{3}\left(\mathrm{DA}\left(G\right)\right)≅G$ if and only if $G≅C_{4}$;*
9. *$T_{2}\left(\mathrm{DA}\left(L\left(G\right)\right)\right)≅G$ if and only if $G≅C_{n}$, where n≥5 and n is odd;*
10. *$T_{2}\left(\mathrm{DA}\left(G\right)\right)≅G$ if and only if $G≅C_{n}$, where n≥5 and n is odd;*
11. *$T_{3}\left(\mathrm{DA}\left(L\left(G\right)\right)\right)≅G$ if and only if $G≅C_{n}$, where n≥7 and 3∤n;*
12. *$T_{3}\left(\mathrm{DA}\left(G\right)\right)≅G$ if and only if $G≅C_{n}$, where n≥7 and 3∤n;*
13. *A(DA(L(G)))≅G if and only if $G≅C_{n}$, where n is odd;*
14. *$D_{2}\left(\mathrm{DA}\left(L\left(G\right)\right)\right)≅\bar{G}$ if and only if $G≅C_{4}$;*
15. *$D_{3}\left(\mathrm{DA}\left(L\left(G\right)\right)\right)≅\bar{G}$ if and only if $G≅C_{3}$;*
16. *$T_{2}\left(\mathrm{DA}\left(L\left(G\right)\right)\right)≅\bar{G}$ if and only if $G≅C_{3}$;*
17. *$T_{3}\left(\mathrm{DA}\left(L\left(G\right)\right)\right)≅\bar{G}$ if and only if $G≅C_{3}$.*
18. *$A\left(\mathrm{DA}\left(L\left(G\right)\right)\right)≅\bar{G}$ if and only if $G≅C_{3},C_{4}$ or $C_{5}$;*
19. *$D_{2}\left(\mathrm{DA}\left(G\right)\right)≅L\left(G\right)$ if and only if $G≅C_{n}$;*
20. *$D_{3}\left(\mathrm{DA}\left(G\right)\right)≅L\left(G\right)$ if and only if $G≅C_{4}$;*
21. *$T_{2}\left(\mathrm{DA}\left(G\right)\right)≅L\left(G\right)$ if and only if $G≅C_{n}$, where n≥5 and n is odd;*
22. *$T_{3}\left(\mathrm{DA}\left(G\right)\right)≅L\left(G\right)$ if and only if $G≅C_{n}$, where n≥7, n is odd and 3∤n;*
23. *A(DA(G))≅L(G) if and only if $G≅C_{n}$, where n is odd;*
24. *$D_{3}\left(\mathrm{DA}\left(G\right)\right)≅\bar{L(G)}$ if and only if $G≅C_{3}$ or $C_{5}$;*
25. *$T_{3}\left(\mathrm{DA}\left(G\right)\right)≅\bar{L(G)}$ if and only if $G≅C_{3}$ or $C_{5}$;*
26. *$A\left(\mathrm{DA}\left(G\right)\right)≅\bar{L(G)}$ if and only if $G≅C_{4}$ or $C_{5}$.*

**Lemma** **3.** *Consider the graph $C_{3}\left(r,s,0\right)$, where r≥1 and s≥0. Then,*

1. *$\bar{C_{3}\left(r,0,0\right)}≅\left(K_{r,s}-\left\{e\right\}\right)\cup K_{1}$, where e is any edge in $K_{r,s}$.*
2.
$\mathrm{DA}\left(C_{3}\left(r,s,0\right)\right)=\left\{\begin{array}{cc} K_{2,r}\cup K_{1}, & \mathrm{if}\;r\geq 1\;\mathrm{and}\;s=0;\\ K_{r,s}\cup 3K_{1}, & \mathrm{if}\;r,s\geq 1. \end{array}\right.$

3. *$D_{2}(\mathrm{DA}\left(C_{3}\left(r,s,0\right)\right)=\left\{\begin{array}{cc} 2K_{2}\cup K_{1}, & \mathrm{if}\;\;r=2\;\mathrm{and}\;s=0;\\ K_{2}\cup K_{1}\cup 3K_{1}, & \mathrm{if}\;\;r=2\;\mathrm{and}\;s=1;\\ K_{2}\cup K_{2}\cup 3K_{1}, & \mathrm{if}\;\;r,s=2;\\ K_{2}\cup 3K_{1}\cup 3K_{1}, & \mathrm{if}\;\;r=2\;\mathrm{and}\;s=3;\\ \left(r+s+3\right)K_{1}, & \mathrm{otherwise}. \end{array}\right.$.*
4. *
$D_{3}(\mathrm{DA}\left(C_{3}\left(r,s,0\right)\right)=\left\{\begin{array}{cc} \bar{K_{4}}, & \mathrm{if}\;\;r=1\;\mathrm{and}\;s=0;\\ K_{2,2}\cup 3K_{1}, & \mathrm{if}\;\;r,s=2;\\ K_{2,3}\cup K_{1}, & \mathrm{if}\;\;r=2\;\mathrm{and}\;s=3;\\ \left(r+s+3\right)K_{1}, & \mathrm{otherwise}. \end{array}\right.$
*
5. *
$\begin{array}{ccc} T_{2}\left(\mathrm{DA}\left(C_{3}\left(r,s,0\right)\right)\right) & = & A\left(\mathrm{DA}\left(C_{3}\left(r,s,0\right)\right)\right)\\ & = & \left\{\begin{array}{cc} K_{r}\cup K_{2}\cup K_{1}, & \mathrm{if}\;\;r\geq 1\;\mathrm{and}\;s=0;\\ K_{r}\cup K_{s}\cup 3K_{1}, & \mathrm{if}\;\;r,s\geq 1. \end{array}\right. \end{array}$
*
6. *
$D_{2}\left(\mathrm{DA}\left(L\left(C_{3}\left(r,s,0\right)\right)\right)\right)=\left\{\begin{array}{cc} K_{2}\cup \bar{K_{2}}, & \mathrm{if}\;\;r=1\;\mathrm{and}\;s=0;\\ \left(r+s+3\right)K_{1}, & \mathrm{otherwise}. \end{array}\right.$
*
7. *
$D_{2}\left(\mathrm{DA}\left(L\left(C_{3}\left(r,s,0\right)\right)\right)\right)=\left\{\begin{array}{cc} K_{4}-K_{2}, & \mathrm{if}\;\;r=1\;\mathrm{and}\;s=0;\\ \left(r+s+3\right)K_{1}, & \mathrm{otherwise}. \end{array}\right.$
*
8. *$T_{3}(\mathrm{DA}\left(C_{3}\left(r,s,0\right)\right)=\left(r+s+3\right)K_{1}$, if r≥1 and s≥0.*

**Lemma** **4.** *Let n≥3 be an integer. Then, $\mathrm{DA}\left(C_{n}\right)≅C_{n}$.*

**Proof.**  The proof follows directly from the definition of a detour antipodal graph. □

**Proposition** **7.** *Let n≥3 be an integer. Then,*

1. *$D_{2}\left(\mathrm{DA}\left(L\left(C_{n}\right)\right)\right)≅C_{n}$, $D_{2}\left(\mathrm{DA}\left(C_{n}\right)\right)≅C_{n}$, $D_{3}\left(\mathrm{DA}\left(L\left(C_{n}\right)\right)\right)≅\bar{C_{n}}$,*
*$T_{2}\left(\mathrm{DA}\left(L\left(C_{n}\right)\right)\right)≅\bar{C_{n}}$, $T_{3}\left(\mathrm{DA}\left(L\left(C_{n}\right)\right)\right)≅\bar{C_{n}}$ if and only if n=3.*
2. *$D_{3}\left(\mathrm{DA}\left(L\left(C_{n}\right)\right)\right)≅G$, $D_{3}\left(\mathrm{DA}\left(C_{n}\right)\right)≅G$, $D_{2}\left(\mathrm{DA}\left(L\left(C_{n}\right)\right)\right)≅\bar{C_{n}}$ if and only if n=4.*
3. *$\mathrm{DA}\left(C_{n}\right)≅\bar{L(C_{n})}$, $\mathrm{DA}\left(L\left(C_{n}\right)\right)≅\bar{C_{n}}$ if and only if n=5.*
4. *$\mathrm{DA}\left(C_{n}\right)≅L\left(G\right)$, $\mathrm{DA}\left(L\left(G\right)\right)≅C_{n}$ for all n≥3.*
5. *$T_{2}\left(\mathrm{DA}\left(L\left(C_{n}\right)\right)\right)≅C_{n}$, $T_{2}\left(\mathrm{DA}\left(C_{n}\right)\right)≅C_{n}$, $T_{2}(\mathrm{DA}\left(C_{n}\right)≅L\left(C_{n}\right)$ if and only if n≥5 and n is odd.*
6. *$T_{3}\left(\mathrm{DA}\left(L\left(C_{n}\right)\right)\right)≅C_{n}$, $T_{3}\left(\mathrm{DA}\left(C_{n}\right)\right)≅C_{n}$, $T_{3}(\mathrm{DA}\left(C_{n}\right)≅L\left(C_{n}\right)$ if and only if n≥7, n is odd and 3∤n.*
7. *$D_{3}(\mathrm{DA}\left(C_{n}\right)≅\bar{L(C_{n})}$, $T_{3}(\mathrm{DA}\left(C_{n}\right)≅\bar{L(C_{n})}$ if and only if n=3,5.*
8. *$A(\mathrm{DA}\left(C_{n}\right)≅\bar{L(C_{n})}$ if and only if n=4,5.*
9. *$A\left(\mathrm{DA}\left(L\left(C_{n}\right)\right)\right)≅\bar{C_{n}}$ if and only if n=3,4,5.*

**Proof.**  Clearly $L\left(C_{n}\right)≅C_{n}$. Note that $\bar{C_{3}}≅\bar{K_{3}}$ and $\bar{C_{4}}≅2K_{2}$. Furthermore, if n≥5, then $\bar{C_{n}}$ is connected and is (n−3)-regular. Thus, the proof follows from Lemmas 1, 2 and 4. □

**Proposition** **8.** *Let G be either connected non-unicyclic or a disconnected graph. Then,*

*$\mathrm{DA}\left(G\right)≇\bar{L(G)}$, DA(G)≇L(G), DA(L(G))≇G, $\mathrm{DA}\left(L\left(G\right)\right)≇\bar{G}$, $D_{2}\left(\mathrm{DA}\left(L\left(G\right)\right)\right)≇G$, $D_{2}\left(\mathrm{DA}\left(G\right)\right)≇G$, $D_{3}\left(\mathrm{DA}\left(L\left(G\right)\right)\right)≇G$, $D_{3}\left(\mathrm{DA}\left(G\right)\right)≇G$, $T_{2}\left(\mathrm{DA}\left(L\left(G\right)\right)\right)≇G$, $T_{2}\left(\mathrm{DA}\left(G\right)\right)≇G$, $T_{3}\left(\mathrm{DA}\left(L\left(G\right)\right)\right)≇G$, $T_{3}\left(\mathrm{DA}\left(G\right)\right)≇G$, A(DA(L(G)))≇G,*

*$D_{2}\left(\mathrm{DA}\left(L\left(G\right)\right)\right)≇\bar{G}$, $D_{3}\left(\mathrm{DA}\left(L\left(G\right)\right)\right)≇\bar{G}$, $T_{2}\left(\mathrm{DA}\left(L\left(G\right)\right)\right)≇\bar{G}$, $T_{3}\left(\mathrm{DA}\left(L\left(G\right)\right)\right)≇\bar{G}$, $A\left(\mathrm{DA}\left(L\left(G\right)\right)\right)≇\bar{G}$, $D_{2}\left(\mathrm{DA}\left(G\right)\right)≇L\left(G\right)$, $D_{3}\left(\mathrm{DA}\left(G\right)\right)≇L\left(G\right)$, $T_{2}\left(\mathrm{DA}\left(G\right)\right)≇L\left(G\right)$, $T_{3}\left(\mathrm{DA}\left(G\right)\right)≇L\left(G\right)$, A(DA(G))≇L(G), $D_{3}\left(\mathrm{DA}\left(G\right)\right)≇\bar{L(G)}$, $T_{3}\left(\mathrm{DA}\left(G\right)\right)≇\bar{L(G)}$, $A\left(\mathrm{DA}\left(G\right)\right)≇\bar{L(G)}$.*

**Proof.**  We need to consider the following cases.

*Case (i):* Let *G* be connected non-unicyclic. Suppose that DA(G)≅L(G). Then, we have |V(G)|=|E(G)|, so *G* is connected and unicyclic, which is a contradiction to our assumption that *G* is non-unicyclic. Hence, DA(G)≇L(G). The proof of the rest of the cases are similar to the above.

*Case (ii):* Let *G* be disconnected. Then, DA(G), DA(L(G)), $T_{2}\left(\mathrm{DA}\left(L\left(G\right)\right)\right)$, $T_{3}\left(\mathrm{DA}\left(L\left(G\right)\right)\right)$, $T_{2}\left(\mathrm{DA}\left(G\right)\right)$, $T_{3}\left(\mathrm{DA}\left(G\right)\right)$ all are totally disconnected. L(G) is disconnected and $\bar{L(G)}$ and $\bar{G}$ are connected. Combining all the information, we get the result.

The proof follows from the above cases. □

**Proposition** **9.** *Let G be a connected unicyclic graph but not a cycle. Then,*

1. *DA(G)≇L(G);*
2. *$\mathrm{DA}\left(G\right)≇\bar{L(G)}$;*
3. *DA(L(G))≇G;*
4. *$\mathrm{DA}\left(L\left(G\right)\right)≇\bar{G}$;*
5. *$D_{2}\left(\mathrm{DA}\left(G\right)\right)≇G$; $D_{3}\left(\mathrm{DA}\left(G\right)\right)≇G$; $T_{2}\left(\mathrm{DA}\left(G\right)\right)≇G$; $T_{3}\left(\mathrm{DA}\left(G\right)\right)≇G$;*
*A(DA(G))≇G; $D_{2}\left(\mathrm{DA}\left(G\right)\right)≇L\left(G\right)$; $D_{3}\left(\mathrm{DA}\left(G\right)\right)≇L\left(G\right)$; $T_{2}\left(\mathrm{DA}\left(G\right)\right)≇L\left(G\right)$; $T_{3}\left(\mathrm{DA}\left(G\right)\right)≇L\left(G\right)$; A(DA(G))≇L(G);*
6. *$D_{2}\left(\mathrm{DA}\left(G\right)\right)≇\bar{G}$; $D_{3}\left(\mathrm{DA}\left(G\right)\right)≇\bar{G}$; $T_{2}\left(\mathrm{DA}\left(G\right)\right)≇\bar{G}$; $T_{3}\left(\mathrm{DA}\left(G\right)\right)≇\bar{G}$;*
*$A\left(\mathrm{DA}\left(G\right)\right)≇\bar{G}$;*
7. *$D_{2}\left(\mathrm{DA}\left(L\left(G\right)\right)\right)≇G$; $D_{3}\left(\mathrm{DA}\left(L\left(G\right)\right)\right)≇G$; $T_{2}\left(\mathrm{DA}\left(L\left(G\right)\right)\right)≇G$; $T_{3}\left(\mathrm{DA}\left(L\left(G\right)\right)\right)≇G$; A(DA(L(G)))≇G;*
8. *$D_{2}\left(\mathrm{DA}\left(L\left(G\right)\right)\right)≇\bar{G}$; $D_{3}\left(\mathrm{DA}\left(L\left(G\right)\right)\right)≇\bar{G}$;*
9. *$T_{2}\left(\mathrm{DA}\left(L\left(G\right)\right)\right)≇\bar{G}$; $T_{3}\left(\mathrm{DA}\left(L\left(G\right)\right)\right)≇\bar{G}$; $A\left(\mathrm{DA}\left(L\left(G\right)\right)\right)≇\bar{G}$;*
10. *$D_{2}\left(\mathrm{DA}\left(G\right)\right)≇\bar{L(G)}$; $D_{3}\left(\mathrm{DA}\left(G\right)\right)≇\bar{L(G)}$; $T_{2}\left(\mathrm{DA}\left(G\right)\right)≇\bar{L(G)}$; $T_{3}\left(\mathrm{DA}\left(G\right)\right)≇\bar{L(G)}$; $A\left(\mathrm{DA}\left(G\right)\right)≇\bar{L(G)}$.*

**Proof.** 

(1): Clearly, L(G) is connected. We show that DA(G) is disconnected. Let Ddiam(G)=k. Then, there exist two vertices $v_{r}$, $v_{s}$ in *G* such that $D_{G}\left(v_{r},v_{s}\right)=k$. Since $v_{r},v_{s}$ are at detour diametrical distance, at least one of them must be pendent; without loss of generality, we assume that $v_{r}$ is pendent. Let $v_{x}$ be a neighbor of $v_{r}$ in *G*. We claim that $v_{x}$ is an isolated vertex in DA(G). Suppose $v_{x}$ is adjacent to any vertex $v_{y}$ in DA(G), then $D_{G}\left(v_{x},v_{y}\right)=k$. Thus, $D_{G}\left(v_{r},v_{y}\right)=k+1$, which is a contradiction to our assumption that the detour diameter of *G* is *k*. Therefore, $v_{x}$ is an isolated vertex in DA(G). It follows that DA(G) is disconnected. Thus, DA(G)≇L(G).
(2): If $G≅C_{3}\left(r,0,0\right)$, r≥1, then $\bar{L(G)}≅K_{1,r}\cup \bar{K_{2}}$. If $G≅C_{3}\left(r,s,0\right)$, r,s≥1, $\bar{L(G)}$ has exactly one pendent vertex. By Lemma 3 (ii), we have $\mathrm{DA}\left(G\right)≇\bar{L(G)}$.
  If *G* is the graph other than these graphs, then by the argument of part (a), DA(G) is disconnected. Thus, $\bar{L(G)}$ is connected. Hence, $\mathrm{DA}\left(G\right)≇\bar{L(G)}$.
(3): If $G≅C_{n}\left(r_{1},r_{2},\ldots ,r_{k}\right)$, then DA(G) has $C_{k+r_{1}+\ldots +r_{m}}$ as a subgraph. Thus, DA(L(G))≇G.
  Now, we assume that *G* is non-isomorphic to the above-mentioned graph. Then, by the similar argument used in the proof of part (1), we get DA(L(G))≇G.
(4): By part (3), DA(L(G)) is disconnected; but $\bar{G}$ is connected. Hence $\mathrm{DA}\left(L\left(G\right)\right)≇\bar{G}$.
(5): By part (1), we have DA(G)) is disconnected. Thus, $D_{2}\left(\mathrm{DA}\left(G\right)\right)$, $D_{3}\left(\mathrm{DA}\left(G\right)\right)$, $T_{2}\left(\mathrm{DA}\left(G\right)\right)$, $T_{3}\left(\mathrm{DA}\left(G\right)\right)$ and A(DA(G)) all are disconnected. However, *G* and L(G) are connected. Thus, none of the above graphs is isomorphic to *G* or L(G).
(6): By part (1) and Lemma 3, none of the graphs $D_{2}\left(\mathrm{DA}\left(G\right)\right)$, $D_{3}\left(\mathrm{DA}\left(G\right)\right)$, $T_{2}\left(\mathrm{DA}\left(G\right)\right)$, $T_{3}\left(\mathrm{DA}\left(G\right)\right)$ and A(DA(G)) is isomorphic to $\bar{G}$.
(7): If $G≅C_{n}\left(r_{1},R_{2},\ldots ,r_{k}\right)$, then $\mathrm{DA}\left(L\left(G\right)\right))≅C_{k+r_{1}+\ldots +r_{m}}$. Thus, $D_{2}(\mathrm{DA}\left(L\left(G\right)\right)$ and $D_{3}(\mathrm{DA}\left(L\left(G\right)\right)$ are totally disconnected. Hence, both $D_{2}(\mathrm{DA}\left(L\left(G\right)\right)$ and $D_{3}(\mathrm{DA}\left(L\left(G\right)\right)$ are not isomorphic to *G*. Furthermore, $T_{2}(\mathrm{DA}\left(L\left(G\right)\right)$, $T_{3}(\mathrm{DA}\left(L\left(G\right)\right)$ and A(DA(L(G)) have either at least two cycles or a cycle as a proper subgraph. However, *G* is unicyclic. Hence, none of $T_{2}(\mathrm{DA}\left(L\left(G\right)\right)$, $T_{3}(\mathrm{DA}\left(L\left(G\right)\right)$ and A(DA(L(G)) is isomorphic to *G*. By part (3), we have DA(L(G)) is disconnected. Thus, none of the graphs $D_{2}(\mathrm{DA}\left(L\left(G\right)\right)$, $D_{3}(\mathrm{DA}\left(L\left(G\right)\right)$, $T_{2}(\mathrm{DA}\left(L\left(G\right)\right)$, $T_{3}(\mathrm{DA}\left(L\left(G\right)\right)$ and A(DA(L(G)) is isomorphic to *G*.
(8): If $G≅C_{n}\left(r_{1},R_{2},\ldots ,r_{k}\right)$, then $\mathrm{DA}\left(L\left(G\right)\right))≅C_{k+r_{1}+\ldots +r_{m}}$ as a subgraph.
  Thus, $D_{2}(\mathrm{DA}\left(L\left(G\right)\right)$ and $D_{3}(\mathrm{DA}\left(L\left(G\right)\right)$ are totally disconnected; however, $\bar{G}$ is not totally disconnected. Thus, both $D_{2}(\mathrm{DA}\left(L\left(G\right)\right)$ and $D_{3}(\mathrm{DA}\left(L\left(G\right)\right)$ are not isomorphic to $\bar{G}$.
  By part (3), we have DA(L(G)) is disconnected. However, $\bar{G}$ is connected. So both $D_{2}(\mathrm{DA}\left(L\left(G\right)\right)$ and $D_{3}(\mathrm{DA}\left(L\left(G\right)\right)$ are not isomorphic to $\bar{G}$.
(9): If $G≅C_{3}\left(r,0,0\right)$, r≥1, $D_{2}(\mathrm{DA}\left(L\left(G\right)\right)$ and $D_{3}(\mathrm{DA}\left(L\left(G\right)\right)$ are totally disconnected, since $T_{2}(\mathrm{DA}\left(L\left(G\right)\right)$, $T_{3}(\mathrm{DA}\left(L\left(G\right)\right)$ and A(DA(L(G)) have no isolated vertices. However, $\bar{G}$ has one isolated vertex. Thus, none of $D_{2}(\mathrm{DA}\left(L\left(G\right)\right)$, $D_{3}(\mathrm{DA}\left(L\left(G\right)\right)$, $T_{2}(\mathrm{DA}\left(L\left(G\right)\right)$, $T_{3}(\mathrm{DA}\left(L\left(G\right)\right)$ and A(DA(L(G)) is isomorphic to $\bar{G}$.
  By part (3), we have DA(L(G)) is disconnected; however, $\bar{G}$ is connected. Hence, none of $D_{2}(\mathrm{DA}\left(L\left(G\right)\right)$, $D_{3}(\mathrm{DA}\left(L\left(G\right)\right)$, $T_{2}(\mathrm{DA}\left(L\left(G\right)\right)$, $T_{3}(\mathrm{DA}\left(L\left(G\right)\right)$ and A(DA(L(G)) is isomorphic to $\bar{G}$.
(10): If $G≅C_{3}\left(r,0,0\right)$ or $C_{3}\left(r,s,0\right)$, r,s≥1, then $\bar{L(G)}$ has either two isolated vertices or exactly one isolated vertex. By Lemma 3, we have $D_{2}\left(\mathrm{DA}\left(G\right)\right)$, $D_{3}\left(\mathrm{DA}\left(G\right)\right)$, $T_{2}\left(\mathrm{DA}\left(G\right)\right)$, $T_{3}\left(\mathrm{DA}\left(G\right)\right)$ and A(DA(G)) are not isomorphic to $\bar{L(G)}$.
  If *G* is the graph other than these graphs, then by the argument of part (a), DA(G) is disconnected. Thus, $\bar{L(G)}$ is connected. Hence, none of $D_{2}\left(\mathrm{DA}\left(G\right)\right)$, $D_{3}\left(\mathrm{DA}\left(G\right)\right)$, $T_{2}\left(\mathrm{DA}\left(G\right)\right)$, $T_{3}\left(\mathrm{DA}\left(G\right)\right)$ and A(DA(G)) is isomorphic to $\bar{L(G)}$.

□

Combining Propositions 7–9, we get the proof of Theorem 3.

## 5. Conclusions

Given a set of wireless sensor nodes and connections, graph theory provides a useful tool to simplify the many moving parts of dynamic systems. In this work, we mainly focused on the study of detour distance graph equations. In particular, we solved some graph equations involving detour two-distance graphs, detour three-distance graphs, detour antipodal graphs and line graphs. This solution is believed to be useful for many researchers and businesses working in wireless sensor networks.

## Data Availability

Not applicable.
